# Impact of Triple Therapy in Elderly Patients with Atrial Fibrillation Undergoing Percutaneous Coronary Intervention

**DOI:** 10.1371/journal.pone.0147245

**Published:** 2016-01-25

**Authors:** Antonia Sambola, Maria Mutuberría, Bruno García del Blanco, Albert Alonso, José A. Barrabés, Héctor Bueno, Fernando Alfonso, Angel Cequier, Javier Zueco, Oriol Rodríguez-Leor, Pilar Tornos, David García-Dorado

**Affiliations:** 1 Cardiology Department, Hospital Universitari Vall d’Hebron, Universitat Autònoma de Barcelona, Barcelona, Spain; 2 Cardiology Department, Hospital General Universitario Gregorio Marañón, Madrid, Spain; 3 Cardiology Department, Hospital Universitario Clínico San Carlos, Madrid, Spain; 4 Cardiology Department, Hospital Universitari de Bellvitge, L’Hospitalet del Llobregat, Barcelona, Spain; 5 Cardiology Department, Hospital Universitario Marqués de Valdecilla, Santander, Spain; 6 Cardiology Department, Hospital Universitari Germans Trias i Pujol, Badalona, Barcelona, Spain; Medstar Washington Hospital Center, UNITED STATES

## Abstract

**Background and Purpose:**

Selecting an ideal antithrombotic therapy for elderly patients with atrial fibrillation (AF) undergoing percutaneous coronary intervention (PCI) can be challenging since they have a higher thromboembolic and bleeding risk than younger patients. The current study aimed to assess the efficacy and safety of triple therapy (TT: oral anticoagulation plus dual antiplatelet therapy: aspirin plus clopidogrel) in patients ≥75 years of age with atrial fibrillation (AF) undergoing percutaneous coronary intervention (PCI).

**Methods:**

A prospective multicenter study was conducted from 2003 to 2012 at 6 Spanish teaching hospitals. A cohort study of consecutive patients with AF undergoing PCI and treated with TT or dual antiplatelet therapy (DAPT) was analyzed. All outcomes were evaluated at 1-year of follow-up.

**Results:**

Five hundred and eighty-five patients, 289 (49%) of whom were ≥75 years of age (79.6±3.4 years; 33% women) were identified. TT was prescribed in 55.9% of patients at discharge who had a higher thromboembolic risk (CHA_2_DS_2_VASc score: 4.23±1.51 vs 3.76±1.40, p = 0.007 and a higher bleeding risk (HAS-BLED ≥3: 88.6% vs 79.2%, p = 0.02) than those on DAPT. Therefore, patients on TT had a lower rate of thromboembolism than those on DAPT (0.6% vs 6.9%, p = 0.004; HR 0.08, 95% CI: 0.01–0.70, p = 0.004). Major bleeding events occurred more frequently in patients on TT than in those on DAPT (11.7% vs 2.4%, p = 0.002; HR 5.2, 95% CI: 1.53–17.57, p = 0.008). The overall mortality rate was similar in both treatment groups (11.9% vs 13.9%, p = 0.38); however, after adjustment for confounding variables, TT was associated with a reduced mortality rate (HR 0.33, 95% CI: 0.12–0.86, p = 0.02).

**Conclusions:**

In elderly patients with AF undergoing PCI, the use of TT compared to DAPT was associated with reduced thromboembolism and mortality rates, although a higher rate of major bleeding.

## Introduction

Atrial fibrillation (AF) is the most common sustained arrhythmia and is a major independent risk factor for stroke and systemic embolism, particularly in elderly patients in whom it is more disabling [1]. The Screening for Atrial Fibrillation in the Elderly (SAFE) trial showed a 12% prevalence of atrial fibrillation in people aged 75–84, and 16% in people aged 85 or over [2]. In addition, the AF rate is the highest among older people (in the United Kingdom, 56% of the population with AF are aged over 75) and the risks of stroke are also the greatest [3,4]. The clinical scenario in which percutaneous coronary intervention (PCI) is necessary in patients with coronary artery disease (CAD) and concomitant AF poses a common treatment dilemma concerning the selection of an effective and safe antithrombotic strategy. Few observations from clinical trials or observational studies have specifically assessed this high-risk group [5–17]. Nevertheless, guidelines and recent expert consensus reports [18–20] recommend “triple therapy” (TT), i.e., the combination of oral anticoagulation (OAC) plus dual antiplatelet therapy (DAPT) for the prevention of recurrent thromboembolic events in high-risk patients with AF undergoing PCI. The selection of a therapeutic regimen in elderly patients is particularly challenging since they are not only at significantly higher risk for thromboembolic events but also for a higher bleeding risk with aggressive antithrombotic therapies [2,3]. We sought to assess the efficacy and safety of TT in patients ≥75 years old with AF undergoing PCI.

## Methods

A prospective cohort study of consecutive patients with AF undergoing PCI and treated with TT or DAPT was analyzed. The population consisted of two distinct prospective cohorts: the first enrolled patients from January 2003 to December 2006 at 6 Spanish teaching centers [7] and the second cohort was recruited at a single center (University Hospital Vall d'Hebron) between 2007 and 2012. In the latter, 325 (55.6%) patients were included from 2003–2006 and the remaining 260 (44.5%) patients from 2007–2012.

Patients with a pre-existing diagnosis of permanent, persistent or paroxysmal AF and those who developed new-onset AF during their index admission were included. The risk of stroke or systemic embolism in patients with AF was assessed using the CHA2DS2-VASc score [1,2,5]. Bleeding risk in patients with AF was estimated by the HAS-BLED score [1,2,5].

At each participating hospital, demographic and clinical data, CHA2DS2-VASc score, bleeding risk estimated by the HAS-BLED score [1,2,5] as low (<3) or high (≥3), percutaneous coronary intervention details, therapeutic regimen prescribed and its recommended duration after stent implantation were recorded by the local investigator.

Since this was an observational study, decisions as to the type of revascularization performed, type of stent used or choice of antithrombotic therapies at discharge were left to the discretion of the attending cardiologists. In patients discharged with DAPT (aspirin 100 mg once a day and clopidogrel 75 mg once a day), one antiplatelet agent was usually stopped at least 1 month following PCI when a BMS was used, and between 3 and 12 months when a DES was used (64.2% paclitaxel and 35.8% of new generation). All patients treated with OAC received vitamin K antagonists plus DAPT or plus clopidogrel alone following the regimen described previously. Thereafter, patients were followed up as part of the routine clinical practice of each hospital.

Clinical and demographic characteristics, risk factors for thromboembolism, and the use of antithrombotic therapy before PCI and at discharge were collected. Clinical follow-up was carried out by telephone interviews and review of clinical records of patients with hospital readmissions and/or outpatient clinic visits to confirm the ongoing antithrombotic regimen followed after discharge, and ascertain the occurrence of any of those adverse events. Bleeding and thromboembolic events were collected by a predefined questionnaire, and all adverse events with their antithrombotic therapy at the timing of occurrence.

The patterns of antithrombotic strategies chosen in patients ≥75 years according to the CHA2DS2-VASC and HAS-BLED scores and the benefits and risks of the use of TT vs DAPT in patients ≥75 years old were analyzed.

### End-point definitions

The *primary end-point* was defined as the occurrence of any thromboembolism event. *Secondary end-points* were: 1) development of any major bleeding episode (BARC ≥3a) and 2) any cause of death. The composite end-points of major adverse cardiac events (MACEs: death, acute myocardial infarction, stent thrombosis or target vessel failure), and major adverse events (MAEs; MACE, major bleeding, or thromboembolism) were also analyzed.

### Definitions of adverse events

*Stroke* was defined as the sudden onset of a neurologic deficit in an area consistent with the territory of a major cerebral artery, and was categorized as *ischemic*, *hemorrhagic* or *unspecified*. Hemorrhagic transformation was not considered as a hemorrhagic stroke. Intracranial hemorrhage was defined as a hemorrhagic stroke or a subarachnoid or subdural hemorrhage. Stroke was diagnosed by techniques such as brain CT or MRI [21]. *Systemic embolism* was acute vascular occlusion of the limbs or any organ (kidneys, mesenteric arteries, spleen, retina or grafts) and was documented by angiography, surgery, scintigraphy or autopsy. *Any degree of bleeding* (major and minor) was defined according to the classification scheme of TIMI and BARC [22,23]. *Acute myocardial infarction* was defined according to the following the criteria of the ESC/ACCF/AHA/WHF [24] and *stent thrombosis* according to the criteria of The Academic Research Consortium [25].

The two cohort studies comply with the Declaration of Helsinki and were approved by the Institutional Review Boards of all hospitals involved [7]. The current study was approved by the Vall d’Hebron University Institutional Review Board. Prior to participating in this study, each investigator obtained local Ethics Committee approval and patients gave their written informed consent.

### Statistical analysis

Descriptive analysis was made using mean ± standard deviation (SD) and range for continuous variables. Absolute and relative frequencies of patients in each category were analyzed for categorical variables. Comparison of continuous variables between the two treatment groups was made by Student's t-test and by chi-square test for categorical variables.

The probability of adverse events during follow-up for each therapeutic group was calculated by Kaplan-Meier analysis and compared by the Mantel-Cox log-rank test in a multivariate model. In this model, we also included variables that showed a probability value <0.15 in the univariate analysis when patients with and without oral anticoagulation at discharge were compared, as well as all demographic, clinical or procedural variables that were potentially not well balanced. A Cox proportional hazards model was used to assess the association between the therapeutic regimen at discharge and cardiovascular events. A 2-tailed p value <0.05 was considered significant. Statistical analysis was performed using the statistical package SPSS 20.0.

## Results

### Characteristics of patients with AF undergoing PCI in relation to age

Five hundred and eighty-five patients with AF undergoing PCI treated with TT or DAPT were identified, 289 of whom (49.4%) were aged 75 years or older. Elderly patients had a higher prevalence of risk factors and thromboembolic and bleeding risk than younger patients. By definition, all elderly patients had a CHA2DS2VASc score≥2. Despite the differences in score results, the use of both antithrombotic strategies was similar between age groups (Table 1).

**Table 1:** Characteristics of patients with atrial fibrillation undergoing percutaneous intervention with regard to age.

	Total patients (N = 585)	Age <75 year (N = 296)	Age≥ 75 years (N = 289)	p value
Women, No. (%)	24.4	16.2	32.9	0.0001
Age, years, mean±SD	66.8±6.2	66.9±6.1	79.6±3.4	0.0001
Smoking, (%)	50.1	57.4	42.9	0.0001
Hypertension, (%)	74.7	70.6	78.9	0.01
Diabetes, (%)	37.6	40.5	34.6	0.08
History of heart failure, (%)	24.9	22.8	27	0.13
History of stroke or embolism, (%)	14.5	10.8	18.3	0.007
Renal failure, (%)	16.2	12.6	19.7	0.01
Peripheral arterial disease, (%)	13.6	12.6	14.6	0.27
COPD, (%)	19	16.5	21.5	0.07
Previous PCI, (%)	32.7	32.7	33.6	0.52
History of CABG, (%)	11.1	10.6	11.6	0.40
Previous MI, (%)	33.6	30.7	36.6	0.07
Acute coronary syndrome, (%)	73.2	70.0	76.5	0.039
CHADS_2_VASC_2_, mean±SD	2.4±1.4	2.4±1.4	4.0±1.4	0.0001
CHADS_2_VASC_2_≥2, (%)	73.2	52.7	100	0.0001
HAS-BLED≥3, (%)	69.8	15.6	84.4	0.02
Patient with DES, (%)	40.2	41.8	38.5	0.22
TT: Coumarin+ Aspirin + Clopidogrel, (%)	45.5	45.9	45	0.44

CHA2DS2VAS-c score indicates: congestive heart failure, hypertension, age ≥75 years, diabetes, history of previous stroke, vascular disease, age 65 to 74 years and sex (female). HAS-BLED indicates: hypertension, renal/liver failure, stroke, bleeding history of predisposition, INR lability, age > 65 years, concomitant drugs or alcohol. COPD: chronic obstructive pulmonary disease; PCI: Percutaneous coronary intervention; MI: myocardial infarction; DES: Drug- eluting stent; TT: Triple therapy.

An acute coronary syndrome was the index event in 76% of elderly patients. A DES was implanted in 38.5% of patients. In those who received TT, 35.8% of DES were new generation. Overall, 159 (55.9%) patients received TT at discharge. The Time in Therapeutic Range (TTR) could only be obtained in patients from our center, and was analyzed in 53.5% of patients ≥ 75 years of age and 48.1% of patients under 75. In those cases, TTR was 68% in patients ≥75 year and 67% of those < 75 years. Among patients treated with DES, the duration of treatment was significantly shorter in those who received TT than in those receiving DAPT (5.7 ± 3.8 vs 7.8 ± 4.2 months, p = 0.007), while for patients who received BMS, the recommended duration of treatment was slightly longer for patients on TT than for those on DAPT (2.9 ± 1.1 vs 1.5 ± 0.5 months, p = 0.04).

### Relationship between of CHA2DS2VASc and HAS-BLED Scores with choice of triple therapy in elderly patients

Baseline characteristics according to treatment group are shown in Table 2. Among 289 elderly patients, the absence of renal failure, previous PCI and ACS as the reason for index admission were associated with a higher use of TT, while age, gender or use of DES had no influence. Patients who received TT had a higher CHA2DS2-VASc score (4.23 ± 1.51 vs 3.76 ± 1.40, p = 0.007) and more frequently a high bleeding risk (HAS-BLED score ≥3: 88.6% vs 79.2%, p = 0.02) than those who received DAPT.

**Table 2:** Characteristics of patients with atrial fibrillation ≥75 years of age undergoing coronary stenting in relation to treatment.

	Total patients (N = 289)	Triple therapy (N = 159)	Dual antiplatelet therapy (N = 130)	p value
Women, No. (%)	32.9	34.6	30.8	0.28
Age, years, mean±SD	79.6±3.4	79.4±3.3	79.8±3.6	0.34
Smoking, (%)	42.9	39.9	46.5	0.15
Hypertension, (%)	78.9	84.3	72.3	0.08
Diabetes, (%)	34.6	34.6	34.6	0.54
History of heart failure, (%)	26.6	26.8	26.3	0.51
History of stroke or embolism, (%)	18.3	21.4	14.6	0.09
Renal failure, (%)	19.7	15.7	24.6	0.04
Peripheral arterial disease, (%)	14.6	11.9	18.0	0.10
COPD, (%)	21.5	18.6	25.0	0.12
Previous PCI, (%)	32.6	42.0	21.1	0.0001
History of CABG, (%)	11.6	12.1	10.9	0.45
Previous MI, (%)	36.6	38.6	34.1	0.25
Acute coronary syndrome, (%)	76.5	72.3	81.5	0.04
CHADS_2_VASc_2_, mean±SD	4.02±1.4	4.23±1.5	3.76±1.4	0.007
CHADS_2_VASc_2_> = 2, (%)	94.1	93.7	94.6	0.47
HAS-BLED≥3, (%)	88.4	88.6	79.2	0.02
Patients with DES, (%)	38.5	36.1	41.4	0.21

CHA2DS2-VASc score indicates: congestive heart failure, hypertension, age ≥75 years, diabetes, history of previous stroke, vascular disease, age 65 to 74 years and sex (female); HAS-BLED indicates: hypertension, renal/liver failure, stroke, bleeding history of predisposition, INR lability, age > 65 years, concomitant drugs or alcohol; COPD: chronic obstructive pulmonary disease; PCI: Percutaneous coronary intervention; MI: myocardial infarction; ACS: acute coronary syndrome; DES: Drug- eluting stent; TT: Triple therapy.

### Triple therapy and 1-year outcomes in elderly patients

Outcomes during follow-up in both groups are shown in Table 2.

One-hundred and six adverse events occurred. On average, TT was used in patients who received BMS and DES for 94.8 ± 71.33 and 188 ± 100 days, respectively (p = 0.0001). However, in patients ≥75 years old who were on TT, no significant differences were found between those patients who received BMS or DES regarding the incidence of stroke or systemic embolism (1% vs 0%, p = 0.63) or bleeding events (total bleeding: 23.8% vs 24.6%, p = 0.52). However, overall, the incidence of thromboembolic events was lower in patients receiving TT compared with those treated with DAPT (0.6% vs 6.9%, p = 0.03). By contrast, patients who received TT tended a trend to have a higher bleeding rate than those treated with DAPT (23.9% vs 16.2%, p = 0.06) owing to an excess of major bleeding (11.7% vs 2.4%, p = 0.002). Eight of the 21 patients who presented major bleeding had an intracraneal hemorrhage and 3 died from this cause. However, no significant differences were found in any cause of death, cardiovascular mortality, MACE and MAE between TT and DAPT (Table 3).

**Table 3:** Comparison of outcomes during 1-year follow-up in patients ≥ 75 years according to treatment.

	Total patients (N = 289)	Triple therapy (N = 159)	Dual antiplatelet (N = 130)	p value
Stroke, (%)	2.4	0.6	4.6	0.03
Stroke or embolism, (%)	3.5	0.6	6.9	0.004
Stent thrombosis, (%)	1.4	1.3	1.6	0.59
Ischemic events, (%)	10.4	9.4	11.5	0.34
BARC <3, (%)	20.4	23.9	16.2	0.06
BARC ≥ 3a, (%)	6.6	11.7	2.4	0.002
Death, (%)	12.8	11.9	13.8	0.38
Cardiovascular death, (%)	8.7	7.5	10.1	0.29
MACE, (%)	19.9	18.2	21.9	0.26
MAE, (%)	36.8	37.1	36.4	0.50

MACE: major adverse cardiovascular event; MAE: major adverse events;

BARC 3a: overt bleeding plus hemoglobin drop of 3 to 5 g/dL* (provided hemoglobin drop is related to bleed). Any transfusion with overt bleeding.

**Fig 1:**
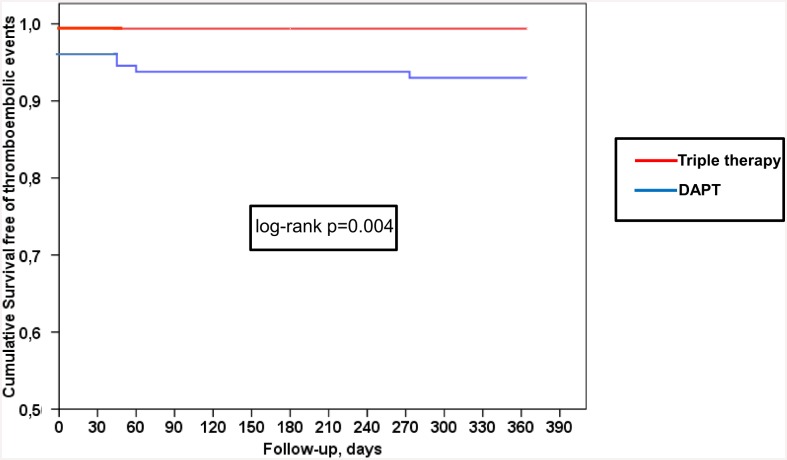
Influence of triple therapy (TT) at discharge in patients ≥75 years. Kaplan-Meier survival curves. Red line, TT used at discharge. Blue line, DAPT used at discharge. Probability of embolic events in patients ≥75 years.

**Fig 2:**
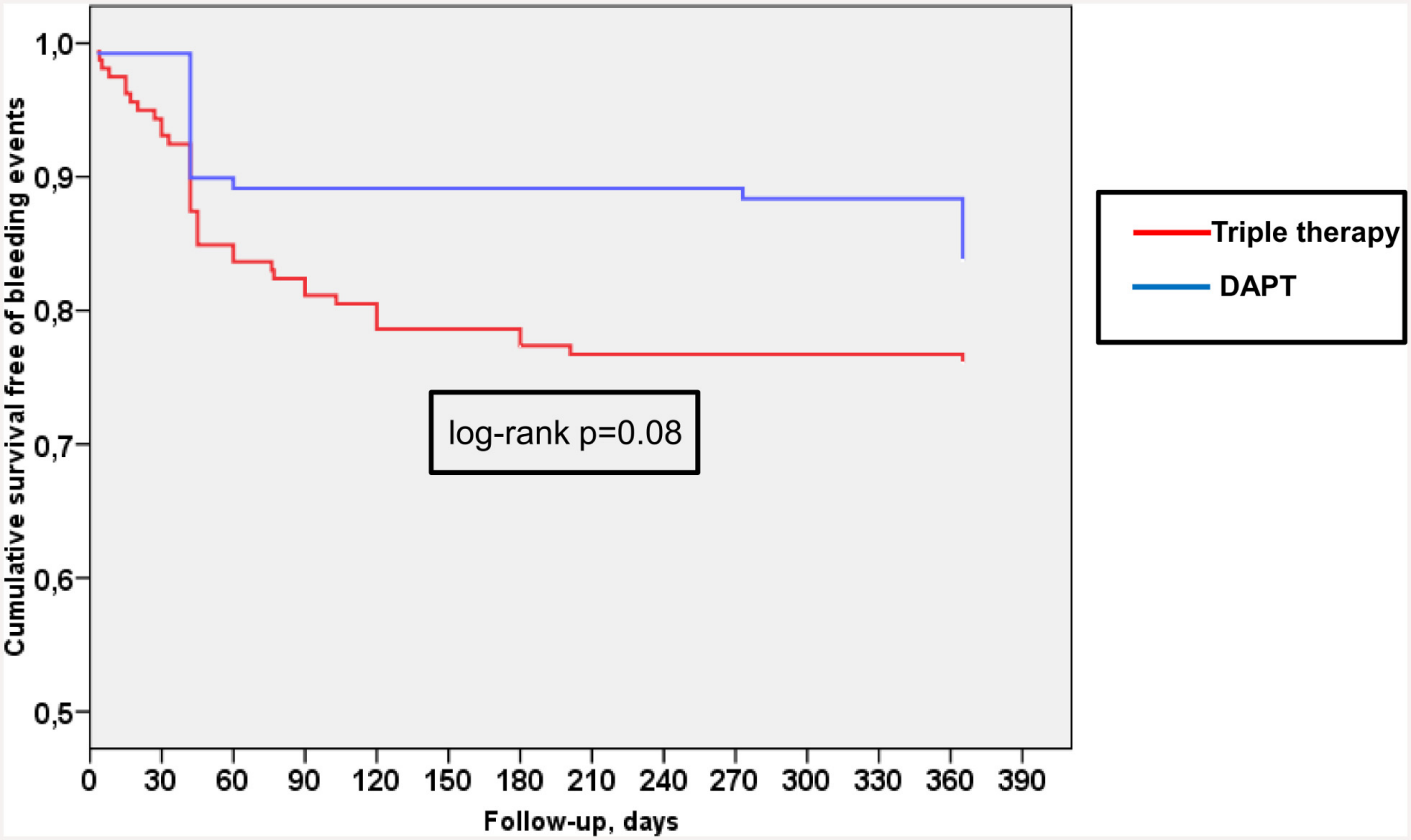
Influence of triple therapy (TT) at discharge in patients ≥75 years. Kaplan-Meier survival curves. Red line, TT used at discharge. Blue line, DAPT used at discharge. Probability of bleeding events in patients ≥75 years.

Survival free of thromboembolic events was significantly higher in patients treated with TT than in those with DAPT (Fig 1, log-rank p = 0.004), but did show a trend towards a higher incidence of major bleeding than patients treated with DAPT (Fig 2, log rank p = 0.08). Survival curves for thromboembolic events diverged very early (before 30 days), but remained quite parallel afterwards, while the curves for bleeding events showed a continuous drop in of events over time (Figs 1 and 2).

CHA2DS2-VASc score was not associated with any adverse event in univariate analysis (thromboembolic events, total bleeding, major bleeding, death from all causes, MACE or MAE). Cox regression analysis selected TT as a protective factor against thromboembolism (HR 0.08, 95% CI 0.01–0.70; p = 0.004) as well as of death from all causes (HR 0.33; 95% CI 0.12–0.86, p = 0.02). In contrast, TT and HAS-BLED score ≥3 were associated with a higher risk of major bleeding events, while renal failure, peripheral vascular disease and heart failure were associated with death from all causes. Finally, number of vessels and renal failure were associated with occurrence of MACE, while TT was not (S1 Table).

## Discussion

This is the first prospective study to specifically show that TT reduced thromboembolic events and all-cause mortality in elderly patients with AF undergoing PCI, despite of the increased rate of bleeding events.

In contrast to previous studies, we observed that the choice of antithrombotic therapeutic strategies, in this particular setting, seems be guided by thromboembolic risk. In our cohort, patients who received TT at discharge had a higher thromboembolic risk than those who received DAPT, in contrast to other authors who found no relationship between the antithrombotic strategy chosen and thromboembolic risk [26–28]. In this regard, Mennuni et al found the choice of the intensity of antithrombotic therapy to be correlated with the risk of ischemic rather than bleeding events in this cohort of patients with AF who underwent PCI [28]. In our cohort, both thromboembolic and bleeding risks were higher in patients who received TT. Since both risks overlap when the CHADS2VASC2 score is used to estimate the thromboembolic risk instead of the CHAD2S risk.

Moreover, on the one hand, the use of TT was significantly higher than observed by other authors. Fosbol et al found a very low rate of TT use (27%) in AF patients ≥65 years of age with non-ST segment elevation myocardial infarction undergoing PCI [26,27], and Shireman et al, observed that, in patients with AF, the use of concomitant warfarin—antiplatelet therapy declined with advancing age [29].

On the other hand, Fosbol et al, surprisingly, found no differences in the bleeding readmission rate between TT compared with DAPT treatment, maybe reflecting a degree of selection bias for warfarin use in patients with low comorbidity [26,27]. However, in agreement with our data, Shireman et al found an increased rate of major bleeding in patients on TT [29].

Our results differ from those reported by Hess et al in that we found a reduction in the incidence of stroke and systemic embolism in elderly patients treated with TT compared with those receiving DAPT. Overall, patients aged ≥75 years have an estimated thromboembolic risk far higher than patients <75 years, and this risk is intrinsically linked to their higher age that by itself counts 2 points on the CHAD2SVAS2C score. Thus, the reasons why elderly patients treated with DAPT had the same embolic incidence as those on TT in Hess’s study are difficult to understand. A possible explanation, perhaps related to the time of enrollment, is that the currently recommended CHAD2SVAS2C and HAS-BLED scores were not used in that study [30]. On the other hand, it is surprising lower prescription of TT (27.1%) in these patients with high thromboembolic risk, when since ever, clinical practice guidelines have recommended the use of TT in patients with AF undergoing PCI regardless of their age. Moreover, that study, despite the large number of data collected, was retrospective, with all the limitations of a study of this nature [30].

Although there is robust evidence supporting the efficacy of OAC in AF, with vitamin K antagonists reducing the stroke risk by up to 3-fold, in routine practice, less than half of elderly patients with AF deemed eligible for warfarin are actually receiving it.[28–31]. Furthermore, new anticoagulants (NOACs) may have contributed to improving the use of anticoagulants in this population; however, until recently their use had not been recommended in patients with AF undergoing PCI [19].

In our study, survival curves for thromboembolic events diverged very early (before 30 days), but remained quite parallel afterwards, while the curves for bleeding events showed a continuous drop in events over time. The different timings of thromboembolic events mostly occurring soon after PCI and bleeding events being more evenly distributed over time suggests a differential time-dependent risk/benefit benefit/risk ratio of antithrombotic therapy. This finding is consistent with the notion that a more intensive antithrombotic therapy, namely TT, may be justified early after PCI even in older patients who have a higher bleeding risk, while longer TT duration of more than 4 weeks may not be justified, as pointed out in guidelines [19,32].

The impact of the warfarin-DAPT combination on bleeding risk remains unclear owing to the inconsistencies between randomized trial designs and clinical practice. These include the exclusion from randomized trials of many elderly patients and those with a higher bleeding risk. Thus, current evidence is insufficient to support clinical decision-making for AF patients requiring both OAC and DAPT.

A recent clinical trial, The Woest, showed that the combination of OAC and clopidogrel might be an option in elderly patients with AF undergoing PCI, since this combination reduces the incidence of bleeding events and does not increase the rate of thrombotic events [17]. However, we believe that the omission of aspirin could trigger thrombotic events in patients at high risk for ischemic events (prevalence of ACS: 73.2%) as other authors have pointed out [33]. Specifically, in our cohort, the therapeutic strategy consisting of oral anticoagulation (OAC) plus clopidogrel was chosen in few patients (50 patients, 7.9%, of whom 23 [54%] were ≥75 years). These small numbers may be explained by the fact that during the study period, this strategy was not accepted after stenting, because of the notion that the omission of DAPT could determine a greater incidence of early and late stent thrombosis [33]. In contrast, dual antiplatelet therapy (DAPT) seemed an attractive alternative to triple therapy (TT), especially in elderly patients or those with difficulties in OAC control, despite the lack of evidence in support of this strategy. In the present study, we found a trend towards higher rates of stent thrombosis in patients on OAC plus clopidogrel compared with those treated with DAPT or TT (7.4% vs 1.3% vs 1.6%, p = 0.94); however the sample size was too small to draw any conclusions.

It is currently impossible to extrapolate the results of the ACS trials in no AF patients to patients with AF and ACS, and an improved assessment of the role of new oral anticoagulants (NOACs: Non-vitamin K-antagonist oral anticoagulants) in AF patients with ACS and PCI will be obtained from ongoing prospective trials.

In summary, our results showed that the use of TT in elderly patients with AF undergoing PCI was guided by the assessment of thromboembolic risk. Furthermore, TT was associated with an early reduction in thromboembolic and mortality risks in patients ≥75 years at the cost of a progressive increase in major bleeding risk. Our findings have important public health implications. The use of TT in elderly patients with AF undergoing PCI may be recommended, despite increasing the rate of bleeding events. Controlled randomized trials are required to define the best strategy and timing of antithrombotic therapy, specifically in elderly patients with AF undergoing PCI.

### Limitations

This study has some limitations. Although it is one of the largest prospective studies in this population group, the number of patients was small. Despite the lack of statistical adjustment for baseline differences, we did not consider making a propensity-score analysis owing to the small size of the sample. A further limitation is the lack of information on the existence and frequency of switching from one therapy to the other in the follow-up. On the other hand, the use of an antithrombotic therapy regimen was at the discretion of the attending physician and was non-randomized.

## Supporting Information

S1 Table Multivariate Cox regression analyses for prediction of adverse outcomes. (DOC)
