# Supplementary material for: Impact of Triple Therapy in Elderly Patients with Atrial Fibrillation Undergoing Percutaneous Coronary Intervention
Source: PLoS One. 2016 Jan 25;11(1):e0147245. doi: 10.1371/journal.pone.0147245 (PMC4726489; doi:10.1371/journal.pone.0147245)
Supplement: S1 Table — (DOC) [file pone.0147245.s001.doc]

**S1 Table. Multivariate Cox regression analyses for prediction of adverse outcomes**

| **Variables** | **HR** | **95% CI** | **P value** |
| --- | --- | --- | --- |
| **Predictors of thromboembolic events** |  |  |  |
| Previous stroke | 2.86 | 0.79-10.39 | 0.10 |
| Hypertension | 3.23 | 0.39-26.74 | 0.27 |
| Renal failure | 2.54 | 0.82-7.87 | 0.10 |
| Heart failure | 0.19 | 0.02-1.58 | 0.12 |
| Femoral access | 4.47 | 1.36-14.68 | 0.01 |
| Triple therapy | 0.22 | 0.07-0.71 | 0.01 |
| **Predictors of major bleeding** |  |  |  |
| Previous stroke | 0.86 | 0.28-2.50 | 0.78 |
| Previous PTCA | 0.87 | 0.93-1.17 | 0.08 |
| ACS | 0.56 | 0.12-2.55 | 0.46 |
| HAS-BLED | 3.32 | 1.16-9.55 | 0.02 |
| Triple therapy | 3.08 | 1.08-8.70 | 0.03 |
| **Predictors of MACE** |  |  |  |
| Renal failure | 2.49 | 1.42-4.37 | 0.001 |
| Peripheral vascular disease | 1.86 | 0.98-3.53 | 0.05 |
| Previous MI | 1.59 | 0.93-2.71 | 0.08 |
| Heart failure | 1.49 | 0.86-2.57 | 0.15 |
| Vessel number | 1.2 | 1.10-1.31 | 0.0001 |
| Triple therapy | 1.07 | 0.61-1.87 | 0.82 |
| **Predictors of all-cause death** |  |  |  |
| Age | 1.05 | 1.0-1.98 | 0.04 |
| Renal failure | 3.10 | 1.61-5.94 | 0.001 |
| Peripheral vasculopathy | 2.62 | 1.28-5.34 | 0.008 |
| Heart failure | 1.95 | 1.03-3.7 | 0.04 |
| Triple therapy | 0.33 | 0.12-0.86 | 0.02 |

CHA_2_DS_2_VAS-c score indicates: congestive heart failure, hypertension, age ≥75 years, diabe­tes, history of previous stroke, vascular disease, age 65 to 74 years and sex category (female). HAS-BLED indicates: hypertension, renal/liver failure, stroke, bleeding history of predisposition, INR lability, age > 65 years, concomitant drugs or alcohol. COPD: chronic obstructive pulmonary disease; PCI: Percutaneous coronary intervention; MI: myocardial infarction; ACS: Acute coronary syndrome; DES: Drug- eluting stent; TT: Triple therapy.
